# Intelligence

**Published:** 1833-03

**Authors:** 


### INTELLIGENCE.

### KING'S COLLEGE, LONDON.

Professor Jones delivered the introductory lecture to his course on Political Economy, on Wednesday the 27th ult.

Professor Burnett will give two courses of lectures on Botany during the present session; the one an extended and practical, the other a short and popular course. The lecture introductory to both courses will be given on Wednesday the 27th instant, at three P.M.

### ROYAL WESTMINSTER OPHTHALMIC HOSPITAL.

The Report of the Treasurer for the year 1832, with the Surgeon's Report, drawn up by Messrs. Hancock and Bedford, the house-surgeons, has just been published. We are equally gratified and astonished at the very small expenditure which has been incurred during the last year; and, when we consider the great number of patients that have attended, (so many of whom are indebted for the preservation of sight to the skill and attention of the surgeon, Mr. Guthrie, and the assistants he has taken so much pains to instruct in the treatment of the diseases of the eye,) we feel justified in asserting that, in no other similar institution is so much serious affliction alleviated at such trifling cost. Notwithstanding the trouble Mr. Guthrie has taken, by the repeated publication of cases of ophthalmia which have been successfully treated in the hospital by the use of the argentum nitratum ointment, to prove the great and speedy efficacy of this treatment, when it is properly employed, we know there are some surgeons who have never yet tried its effects; and we also know that their patients have much reason to regret their obstinate adherence to preconceived opinions and practice, which ample experience must have shown them are not to be relied upon, and the fallacy of which they might quickly be convinced of by a few visits to this establishment.

The number of patients admitted during the last half-year amounts to 1021, making a total, for 1832, of 1705. Thirty-one operations have been performed for cataract, and all succeeded in restoring good and useful sight. The most remarkable cases are those of Ann Frehorne, a paralytic woman, aged eighty-two; Frederic Herbert, who was born blind, and remained so until he was thirteen years old; and four children, in one family of the name of Griffiths, all of whom were born blind, or nearly so, and restored to sight by operation. The number of operations for cataract has been few, in consequence of its having been thought fit to try a particular and private remedy, supposed, by a gentleman of the name of Marshall, to possess the property of dissolving a cataract in its early stage. Twenty-seven cases were placed under his management, but the results of his treatment are by no means satisfactory; for in no one instance does the report state that any decided benefit was obtained from it.

Several cases are mentioned to show the mischievous results of purulent ophthalmia, when uncontrolled by proper treatment, and the great advantages which are derived from that which is peculiar to this hospital. From our personal experience at this and other hospitals, as well as in private practice, we can add our testimony to the great superiority of the treatment followed by Mr. Guthrie, to that which is too commonly adopted, of large and repeated bleedings.

In every point of view, the Westminster Ophthalmic Hospital deserves the warm support of the public and the profession; and once more we urge practitioners, and more especially those who are medical officers of similar institutions, to take advantage of the opportunities which are here afforded of removing any doubts they may entertain as to the efficacy of this treatment in various cases of ophthalmia, which is tried upon so large a scale, and with such immense benefit to the patients.

---

### MEDICAL EXAMINATIONS AT EDINBURGH.

We understand that a very important, and in our opinion a very desirable, alteration is ordered to be made in the medical examinations in the University of Edinburgh: *they are henceforth to be conducted in the English instead of in the Latin language.* That all medical practitioners should have had a sound classical education, we freely admit: and it appears to us equally desirable that they should be compelled to give sufficient proofs of their classical attainments when they present themselves as candidates for any medical degree: but we must at the same time maintain, the primary object of all medical examinations being to ascertain as fully as possible the professional qualifications of the party examined, that it is utterly absurd to conduct the examination in a language which very few persons indeed can speak with fluency or facility enough to enable them to do justice to themselves in expressing their opinions upon medical topics. It is quite notorious that much more apprehension of failure exists in the mind of most candidates, on account of the difficulty of maintaining a discourse in Latin, than from the fear of not being prepared to give the required and very proper proofs of their medical knowledge; and we believe it cannot be denied that any thing can be more barbarous, or more "innocent of grammatic rule," than the jargon called Latin which often "passes" at medical examinations. Surely, it would be wiser, and much more for the advantage of the public, (for whose benefit such examinations are instituted,) to give the candidate full and free liberty of speech, and to require him to speak his mother-tongue correctly, than to distract his attention from the chief object which should occupy his thoughts, by obliging him to seek for words rather than for ideas. We venture to say, that there is not one man in a hundred who goes through a medical examination conducted in Latin, who can, with such a stumbling-block in his way, express himself to his own satisfaction. And, even supposing that all candidates for medical degrees were compelled to speak the Latin language with perfect purity and correctness, we doubt the advantages of imposing an obligation upon them, to comply with which much time must necessarily have been previously expended, which, *we think,* might have been more profitably employed. There are very few persons who succeed so completely in mastering the difficulties of a living language, even with all the advantage of residence in the country and constant intercourse with the natives, as to enable them to "unlock all their intellectual stores" when they enter into a lengthened conversation in it: the difficulties of speaking a *dead* language are tenfold, and can only be overcome by the application of years. To support the dignity and respectability of the profession, it is proper that its members should be men of a good and even liberal education; but their classical attainments may be sufficiently tested by obliging them to read Latin from any work that may be selected at the moment of examination.

---

### APOTHECARIES' HALL.

*To the Editors of the London Medical and Physical Journal.*

Gentlemen: The enclosed circular letter has already been sent to the principal dispensaries in London; but, as it is impossible to forward it to all such institutions in the country, perhaps you will have the goodness to give it extensive publicity by inserting it in the London Medical and Physical Journal.

I am, gentlemen, your obedient servant,

John Watson.

DISPENSARIES AS SCHOOLS OF PRACTICAL MEDICINE.

*Apothecaries' Hall; Feb.* 7, 1833.

To the Physicians of ——— Dispensary.

Gentlemen: I am instructed by the Court of Examiners to call your attention to the notices relative to dispensaries, which were published in the regulations of the Court in September 1830, and in August 1832; and to remind you, that the time for acting upon them is now arrived.

The Court are ready to receive applications for the recognition of dispensaries, from the physicians attached to these institutions respectively; and, with the view of saving unnecessary trouble to the medical officers of these establishments, the Court have further instructed me to state, that they will recognise, as schools of practical medicine, such dispensaries only as shall give satisfactory evidence on the following points: viz.

"That the dispensary is situated in some city or town in which there is a medical school recognised by the Court.

"That the rules for the government of the dispensary permit the attendance of students, and that the physicians afford them opportunities of acquiring practical knowledge in medicine.

"That the dispensary (if within the limits of the jurisdiction of the Royal College of Physicians of London) is under the medical care of at least two physicians, each of whom is a fellow, candidate, or licentiate of the Royal College; and, if beyond these limits, that it is under the care of at least two physicians, who, if not so qualified, are graduated doctors of medicine of a British university, of four years' standing.

"And that the apothecary of the dispensary is legally qualified, either by having been in practice prior to or on the 1st day of August, 1815, or by having received a certificate of qualification from the Court of Examiners,"

I have the honour to be, gentlemen, your obedient servant,

JOHN WATSON, Secretary.

N. B. Every certificate of attendance is to be signed by all the physicians connected with the dispensary attended by the student; and the Court especially request, that no certificate may be signed unless the pupil has been *uniformly regular* in his attendance.

---

LITERARY INTELLIGENCE.

The First Part of Professor BURNETT'S "Outlines of Botany," which we announced some time since, will be published the latter end of this month; and the succeeding fasciculi as soon as the engravings, which are in a very forward state, will permit.

### METEOROLOGICAL REGISTER,

By Messrs. HARRIS and Co., Mathematical Instrument Makers, 50, High Holborn.

| Jan. | Moon. | Rain gauge. | Thermometer. 9 a.m. | max. | min. | Barometer. 9a.m. | 10p.m. | De Luc's Hygrometer 9a.m. | 10p.m. | Winds. 9 a.m. | 10p.m. | Atmospheric Variation. 9 a.m. | 2 p.m. | 10 p.m. |
|---|---|---|---|---|---|---|---|---|---|---|---|---|---|---|
| 20 | ● | | 37 | 39 | 31 | 30.07 | 30.09 | 75 | 74 | SE | SE | fine | cloudy | fine |
| 21 | | | 34 | 36 | 30 | .10 | .10 | 74 | 74 | ESE | SE | foggy | fine | |
| 22 | | | 31 | 35 | 28 | .17 | .27 | 73 | 74 | SE | SE | | | |
| 23 | | | 30 | 36 | 29 | .36 | .36 | 74 | 75 | SE | SE | | | |
| 24 | | | 35 | 37 | 31 | .25 | .20 | 76 | 78 | SE | ESE | fine | | |
| 25 | | | 34 | 36 | 34 | .02 | 29.95 | 80 | 80 | SE | SE | foggy | | foggy |
| 26 | | | 36 | 40 | 34 | 29.92 | .92 | 80 | 80 | SSE | W | | cloudy | fine |
| 27 | | | 39 | 42 | 36 | .87 | .82 | 80 | 80 | SW | SW | fine | fine | fine |
| 28 | | | 40 | 44 | 40 | .76 | .63 | 80 | 80 | WNW | W | | | rain |
| 29 | ☽ | | 41 | 41 | 38 | .20 | .13 | 80 | 82 | SW | E | showery | cloudy | sleet |
| 30 | | | 40 | 41 | 33 | .32 | .55 | 85 | 83 | NW | NW | foggy | fine | fine |
| 31 | | | 35 | 39 | 35 | .52 | .31 | 83 | 83 | WNW | S | | cloudy | rain |
| Feb. | | | | | | | | | | | | | | |
| 1 | | | 37 | 40 | 36 | .33 | .46 | 82 | 83 | N | SE | | | cloudy |
| 2 | | | 46 | 52 | 41 | 28.80 | 28.68 | 85 | 83 | SE | W | rain | cloudy | fine |
| 3 | | .07 | 45 | 50 | 43 | .86 | 29.14 | 81 | 79 | WNW | WNW | fine | fine | |
| 4 | ○ | | 48 | 54 | 51 | 29.48 | .64 | 78 | 79 | W | W | rain | fine | |
| 5 | | .12 | 54 | 55 | 47 | .68 | .73 | 80 | 82 | W | WSW | foggy | fine | |
| 6 | | | 50 | 54 | 48 | .82 | .66 | 82 | 81 | WSW | SW | | cloudy | cloudy |
| 7 | | | 52 | 55 | 48 | .68 | .75 | 80 | 80 | WNW | WSW | | fine | |
| 8 | | | 53 | 55 | 43 | .52 | .52 | 80 | 80 | WSW | SW | cloudy | | fine |
| 9 | | | 45 | 46 | 42 | .45 | .59 | 80 | 80 | NNE | WNW | rain | | |
| 10 | | | 47 | 52 | 45 | .36 | .25 | 79 | 79 | WSW | SW | rain | rain | |
| 11 | ☾ | .26 | 50 | 53 | 43 | .22 | .22 | 77 | 77 | W | W | fine | fine | |
| 12 | | | 50 | 54 | 42 | .40 | .42 | 77 | 79 | WSW | SW | cloudy | | cloudy |
| 13 | | | 43 | 50 | 46 | .22 | .11 | 79 | 78 | W | WSW | showery | cloudy | rain |
| 14 | | .09 | 48 | 50 | 42 | .09 | .18 | 76 | 76 | W | SW | fine | showery | |
| 15 | | | 43 | 44 | 35 | .05 | .22 | 76 | 75 | N | NW | cloudy | fine | fine |
| 16 | | | 38 | 42 | 37 | .46 | .55 | 75 | 77 | NW | NW | fine | | |
| 17 | | | 42 | 45 | 35 | .42 | .30 | 78 | 82 | W | W | cloudy | sleet | rain |
| 18 | | .14 | 38 | 47 | 38 | .33 | .43 | 79 | 77 | W | NNW | foggy | fine | fine |
| 19 | ● | | 44 | 45 | 42 | .67 | .59 | 75 | 77 | NW | SW | fine | | |

In consequence of the frost in January, the quantity of rain cannot be given for that month.

